# Association of polymorphisms in *TGFB1*, *XRCC1*, *XRCC3* genes and CD8 T-lymphocyte apoptosis with adverse effect of radiotherapy for prostate cancer

**DOI:** 10.1038/s41598-022-25328-6

**Published:** 2022-12-09

**Authors:** Emina Mališić, Nina Petrović, Muriel Brengues, David Azria, Ivana Z. Matić, Ivana Srbljak Ćuk, Katarina Kopčalić, Tatjana Stanojković, Marina Nikitović

**Affiliations:** 1 Department of Experimental Oncology, Institute for Oncology and Radiology of Serbia, Pasterova 14, 11 000 Belgrade, Serbia; 2 “VINČA“ Institute of Nuclear Sciences-National Institute of the Republic of Serbia, University of Belgrade, Belgrade, Serbia; 3 IRCM, INSERM, University Montpellier, ICM, Montpellier, France; 4 Department of Radiation Oncology, Institute for Oncology and Radiology of Serbia, Belgrade, Serbia; 5 Faculty of Medicine, University of Belgrade, Belgrade, Serbia

**Keywords:** Cancer, Molecular biology, Biomarkers, Molecular medicine, Oncology

## Abstract

The genetic background of each person might affect the severity of radiotherapy (RT)-induced normal tissue toxicity. The aim of study was to evaluate the influence of *TGFB1* C-509T and Leu10Pro, *XRCC1* Arg280His and *XRCC3* Thr241Met polymorphisms as well as the level of radiation-induced CD8 T-lymphocyte apoptosis (RILA) on adverse effects of RT for prostate cancer (PCa). The study included 88 patients with localized or locally advanced PCa who were treated with RT. The polymorphisms were determined by PCR–RFLP analysis on DNA from peripheral blood mononuclear cells. RILA values were measured by flow cytometry. We found that CT genotype of *TGFB1* C-509T could be protective biomarker for acute genitourinary (GU) and gastrointestinal (GI) radiotoxicity, while Thr variant of *XRCC3* Thr241Met could predict the risk for acute GU radiotoxicity. Correlation between RILA values and toxicity was not detected. Univariate logistic regression analysis showed that Gleason score and risk group were risk factors for late GU, while for late GI radiotoxicity it was diabetes mellitus type 2. However, in multivariate model those were not proven to be significant and independent risk factors. Identification of assays combination predicting individual radiosensitivity is a crucial step towards personalized RT approach.

## Introduction

About half of all prostate cancer (PCa) patients receive radiotherapy (RT) at some stage of the disease, either as a single curative treatment or as adjuvant/salvage treatment after radical prostatectomy^1^. However, RT is associated with a wide spectrum of side effects (toxicity) in the surrounding malignantly non-transformed tissues. Acute toxicity occurs during the treatment, or shortly after the end of therapy. Usually, it is transient, and reversible, and affects highly dividing tissues, such as the skin and mucosa of the bladder and intestine, resulting in dermatitis, urinary incontinence, cystitis or diarrhea, respectively^2^, whereas late toxicity effects, such as fibrosis, rectal bleeding, a bladder and erectile dysfunction, or even secondary malignancies, occur months to years after treatment and can persist for lifetime^3^. Radiotherapy toxicity may have significant impact on the quality of life of PCa patients because most of them have clinically localized and indolent tumors at diagnosis with a very successful 5-year survival rate of nearly 100%^4^.

The aim of radiogenomics is to find the genetic variants associated with adverse reactions to RT such as single nucleotide polymorphisms (SNPs), gene alteration, copy number variations etc. SNPs make up 90% of germinal variations in DNA sequence^5^. These polymorphic variations may underlie the basis of individual patients' radiosensitivity because they change the thermodynamic stability of mRNA molecules, transcription and translation rates, and/or protein–protein interactions, which may reflect on oxidative balance, contributing to altered DNA damage signaling and cell cycle control. SNPs or gene variants can also change the structure and conformation of DNA repair proteins, members of inflammatory response, and can influence cytokine activity related to fibrosis as well as general metabolism and homeostasis^5^.

Transforming growth factor b1 (TGFβ1), the protein encoded by *TGFB1* gene, is a multifunctional cytokine produced primarily by endothelial, hematopoietic, and connective tissue cells. TGFβ1 regulates various cell functions such as proliferation, differentiation, embryonic development, immune response, wound healing, and angiogenesis^6^. In irradiated cells, TGFβ1 is a mediator of inflammatory response involved in proliferation, differentiation, the production of extracellular matrix proteins and fibrosis^7^.

Previous studies have hypothesized that the *TGFB1* promoter phenotype affects gene transcriptional activity and TGFβ1 plasma levels. The presence of the T allele at − 509 bp of the promoter region (C>T, rs1800469) is associated with higher concentrations of TGFβ1 than C allele^8^. The T version increases the amount of TGFβ1 produced by preventing AP1 from binding to this region whereas in the C version it would normally downregulate production^9^.

The T>C transition at codon 10 in exon 1 of *TGFB* results in leucine to proline substitution (Leu10Pro) (rs1800470). This transition results in increased levels of TGFB1 mRNA and protein in individuals with the proline allele compared to those with the leucine allele^10^.

*XRCC1* (X-ray repair cross complementing group 1) and *XRCC3* (X-ray repair cross complementing group 3) genes encode for proteins that are involved in single-strand DNA breaks and base excision repair and homologous recombination (HR) repair of radiation-induced DNA double-strand breaks (DSBs), respectively^11^. Disruption of these pathways has the potential to affect the normal tissue response to RT. One of the most extensively studied SNP in *XRCC1* is Arg280His in exon 9 (G>A, rs25489). The 280His protein variant is associated with increased repair activity and could be potential biomarker of acute and/or late radiotoxicity^12^. One of the most common SNP investigated in *XRCC3* is Thr241Met in exon 7 (C>T, rs861539). The 241Met protein variant was reported to be associated with elevated levels of DNA adducts^13^, chromosomal deletions^14^, sensitivity to ionizing radiation, and cross-linking agents^15^.

Radiation-Induced Lymphocyte Apoptosis (RILA) is defined as the percentage of apoptosis of peripheral blood lymphocytes (PBLs) irradiated with 8 Gy minus the percentage of apoptosis of control non-irradiated PBLs (0 Gy). No association was found between early toxicity and T-lymphocyte apoptosis levels while radiation-induced T-lymphocyte apoptosis can significantly predict differences in late toxicity between individuals. A negative predictive value was found in the case of high RILA value and a late toxicity grade < 2. On the contrary, all severe side-effects (grade ≥ 2) were observed in patients with low values of RILA^16^. Further studies confirmed that RILA levels can significantly predict the risk for development late radiation-induced toxicity^17,18^.

According to what was previously mentioned, the aim of this study was to examine the impact of polymorphisms *TGFB1* C-509T and Leu10Pro*, XRCC1* Arg280His, *XRCC3* Thr241Met as well as the radiation-induced CD8 T-lymphocyte apoptosis rate on the RT-induced normal tissue toxicity in Serbian PCa patients.

## Results

### Patient', clinical and treatment characteristics

The characteristics of the study cohort and RT treatment are shown in Table 1.

**Table 1:** Patient's, clinical and treatment characteristics.

Mean age ± standard deviation	69.5 years ± 6.6
Initial PSA level	3.9–70 ng/mL (median 12.1 ng/mL)
**Diabetes mellitus type 2**	
Yes	16 (18.2%)
No	72 (81.8%)
**Smoking status**	
Active/former	62 (70.5%)
Non-smokers	26 (29.5%)
**Chronic hypertension**	
Yes	54 (61.4%)
No	34 (38.6%)
**Gleason score, median (min- max)**	7 (6–9)
Risk group	
Low	9 (10.2%)
Intermediate	47 (53.4%)
High	32 (36.4%)
**Type of radiotherapy**	
Radical	50 (56.8%)
Postoperative	38 (43.2%) Adjuvant: 22/38 Salvage: 16/38

PSA- prostate specific antigen.

### Radiation‑induced toxicity description

All 88 patients included in the study were evaluated for toxicity throughout follow-up time (acute and late). Among them, 83 patients (94.3%) experienced acute genitourinary (GU) toxicity (grade 1, 2, and 3: 65.1%, 25.3%, and 9.6% patients, respectively. Eighty (90.9%) patients had acute gastrointestinal (GI) toxicity (grade 1 and 2 in 75% and 25% patients).

Late GU toxicity was developed in 62 (70.5%) patients. Among them, grade 1, 2, and 3 had: 62.9%, 32.3%, and 4.8% patients, respectively. Late GI toxicity appeared in 24 of 88 patients (27.3%) (grade 1 and 2 in 83.3% and 16.7% patients). The follow up period for late GU and GI was 3 to 54 months after RT cessation (median 42 and 39 months, respectively).

### TGFB1, XRCC1 and XRCC3 polymorphisms

The distributions of genotypes of *TGFB1* C-509T, *TGFB1* Leu10Pro, *XRCC1* Arg280His, and *XRCC3* Thr241Met were given in Table 2. The frequencies of alleles were: C (69.8%) and T (30.2%) for *TGFB1* C-509T, Leu (56.7%) and Pro (43.3%) for *TGFB1* Leu10Pro, Arg (98.8%) and His (1.2%), Thr (63.6%) and Met (36.4%). The study group was in Hardy–Weinberg equilibrium in relation to the investigated gene loci.

**Table 2:** The distributions of genotypes and alleles of investigated SNPs in prostate cancer patients.

Genotype	n (%)	Alelle (%)	Hardy–Weinberg equilibrium (*p* value)
***TGFβ1* C-509T**			
CC	45 (52.3%)	C (69.8%)	0.108
CT	30 (34.9%)	T (30.2%)	
TT	11 (12.8%)		
***TGFβ1* Leu10Pro**			
LeuLeu	25 (30.5%)	Leu (56.7%)	0.538
LeuPro	43 (52.4%)	Pro (43.3%)	
ProPro	14 (17.1%)		
***XRCC1* Arg280His**			
ArgArg	81 (97,6%)	Arg (98.8%)	0.911
ArgHis	2 (2.4%)	His (1.2%)	
HisHis	0 (0%)		
***XRCC3* Thr241Met**			
ThrThr	33 (40.7%)	Thr (63.6%)	0.902
ThrMet	37 (45.7%)	Met (36.4%)	
MetMet	11 (13.6%)		

n- Number of patients.

The differences in the distribution of genotypes of *TGFB1* C-509T, *TGFB1* Leu10Pro, and *XRCC3* Thr241Met between patients with or without acute or late RT-induced GU or GI toxicity as well as different grades of toxicity were given in Table 3.

**Table 3:** Distribution of acute and late RT-induced GU or GI toxicity between patients with genotypes of *TGFB1* C-509T, *TGFB1* Leu10Pro and *XRCC3* Thr241Met.

	*TGFB1* C-509T	*TGFB1* Leu10Pro	*XRCC3* Thr241Met
**Acute** **GU toxicity**	CC: 97.8% CT: 86.7% TT: 100%	LeuLeu: 100% LeuPro: 90.7% ProPro: 100%	ThrThr: 100% ThrMet: 94.6% MetMet: 81.8%
**Acute** **GU toxicity** **(grade 1 vs. Grade 2 *plus* 3)**	**Grade 1:** CC: 70.5% CT: 61.5% TT: 54.5% **Grade 2 plus 3:** CC: 29.5% CT: 38.5% TT: 45.5%	**Grade 1:** LeuLeu: 76.0% LeuPro: 59.0% ProPro: 64.3% **Grade 2 plus 3:** LeuLeu: 24.0% LeuPro: 41% ProPro: 35.7%	**Grade 1:** ThrThr: 54.5% ThrMet: 71.4% MetMet: 77.8% **Grade 2 plus 3:** ThrThr: 45.5% ThrMet: 28.6% MetMet: 22.2%
**Acute** **GI toxicity**	CC: 95.6% CT: 83.3% TT: 100%	LeuLeu: 92.0% LeuPro: 88.4% ProPro: 100%	ThrThr: 90.9% ThrMet: 91.9% MetMet: 90.9%
**Acute** **GI toxicity** **(grade 1 vs. Grade 2 plus 3)**	**Grade 1:** CC: 76.7% CT: 68% TT: 81.8% **Grade 2:** CC: 23.3% CT: 32% TT: 18.2%	**Grade 1:** LeuLeu: 78.3% LeuPro: 73.7% ProPro: 78.6% **Grade 2:** LeuLeu: 21.7% LeuPro: 26.3% ProPro: 21.4%	**Grade 1:** ThrThr: 76.7% ThrMet: 76.5% MetMet: 60% **Grade 2:** ThrThr: 23.3% ThrMet: 23.5% MetMet: 40%
**Late** **GU toxicity**	**Yes:** CC: 71.1% CT: 70.0% TT: 63.6%	**Yes:** LeuLeu: 72.0% LeuPro: 72.1% ProPro: 64.3%	**Yes:** ThrThr: 78.8% ThrMet: 64.9% MetMet: 63.6%
**Late** **GU toxicity** **(grade 1 vs. Grade 2 plus 3)**	**Grade 1:** CC: 71.9% CT: 47.6% TT: 71.4% **Grade 2 plus 3:** CC: 28.1% CT: 52.4% TT: 28.6%	**Grade 1:** LeuLeu: 72.2% LeuPro: 58.1% ProPro: 66.7% **Grade 2 plus 3:** LeuLeu: 27,8% LeuPro: 41.9% ProPro: 33.3%	**Grade 1:** ThrThr: 61.5% ThrMet: 70.8% MetMet: 42.9% **Grade 2 plus 3:** ThrThr: 38.5% ThrMet: 29.1% MetMet: 57.1%
**Late** **GI toxicity**	**Yes:** CC: 28.9% CT: 23.3% TT: 36.4%	**Yes:** LeuLeu: 28.0% LeuPro: 27.9% ProPro: 28.6%	**Yes:** ThrThr: 24.2% ThrMet: 32.4% MetMet: 18.2%
**Late** **GI toxicity** **(grade 1 vs. Grade 2 plus 3)**	**Grade 1:** CC: 76.9% CT: 100% TT: 75.0% **Grade 2:** CC: 23.1% CT: 0% TT: 25.0%	**Grade 1:** LeuLeu: 71.4% LeuPro: 91.7% ProPro: 75.0% **Grade 2:** LeuLeu: 28.6% LeuPro: 8.3% ProPro: 25.0%	**Grade 1:** ThrThr: 62.5% ThrMet: 91.7% MetMet: 100% **Grade 2:** ThrThr: 37.5% ThrMet: 8.3% MetMet: 0%

GU- genitourinary; GI- gastrointestinal.

**Figure 1:**
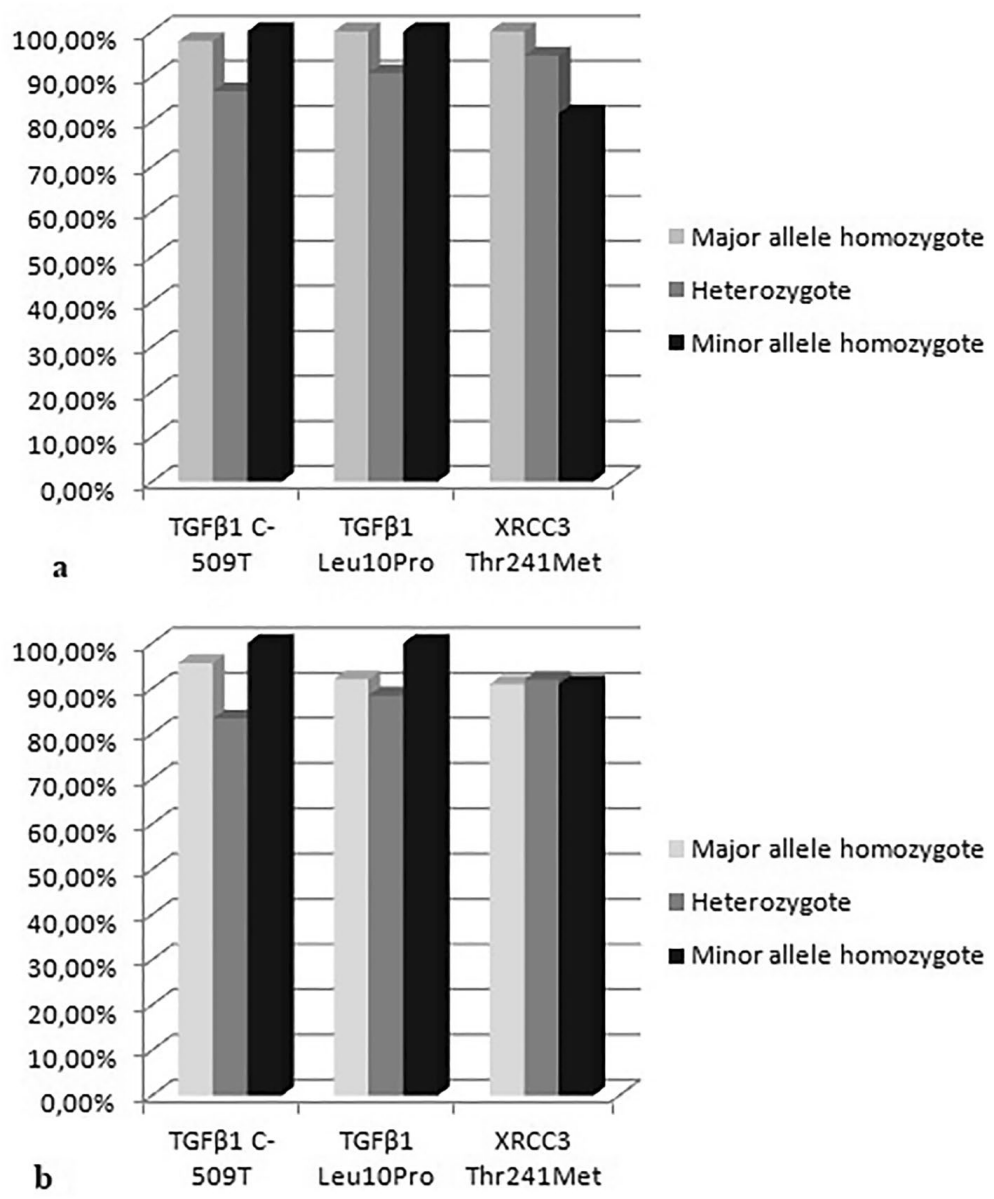
The differences in the distribution of genotypes of *TGFB1* C-509T, *TGFB1* Leu10Pro, and *XRCC3* Thr241Met between patients with (**a)** acute RT-induced GU toxicity and (**b)** acute RT-induced GI toxicity.

Heterozygote carriers of *TGFB1* C-509T (CT) had lower rate of acute GU as well as acute GI toxicity than homozygotes (CC *plus* TT) (Fig. 1a,b) and this difference was statistically significant (*p* = 0.048 and *p* = 0.047, Fisher exact test). Additionally, the OR (95% CI) in the over-dominant model (0.12 (0.01–1.11) for acute GU and 0.19 (0.03–1.02) for acute GI) indicated a lower risk for the development of acute RT-induced toxicity in heterozygote patients. The frequencies of acute GU toxicity grade ≥ 2 arose from CC and CT to TT genotype while in acute GI toxicity the heterozygote patients had higher frequency of grade 2 toxicity than CC and TT genotype but without statistically significant difference in any of the examined genetic models. In late GU toxicity, the CC and CT carriers had slightly higher rate than TT but without statistical significance. However, heterozygote carriers had nearly double higher rate of grade ≥ 2 toxicity than homozygotes. Contrary to late GU toxicity, individuals with TT genotype had higher rate of overall late GI toxicity, and the heterozygotes had lower rate of grade ≥ 2 than homozygotes but these differences did not show statistical significance.

Heterozygote Pca patients of *TGFB1* Leu10Pro (LeuPro) had lower rate of acute GU as well as acute GI toxicity than homozygotes (LeuLeu *plus* ProPro) (Fig. 1a,b). The frequency of acute GU and GI toxicity grade ≥ 2 were higher in LeuPro than homozygote carriers but without statistically significant difference. In late GU toxicity, LeuLeu and LeuPro carriers had slightly higher rate than ProPro. For late GI toxicity, there was a similar frequency of all genotypes of *TGFB1* Leu10Pro. However, heterozygote carriers had higher rate of grade ≥ 2 GU but lower rate of grade ≥ 2 GI toxicity than homozygotes but without statistically significant difference.

We did not perform statistical analysis for *XRCC1* Arg280His polymorphism because only two of 83 analyzed patients had ArgHis genotype and everyone else ArgArg. Both of ArgHis patients had acute GU and GI toxicity grade 1. One of them experienced late GU toxicity grade 1 and the other late GI toxicity grade 1.

There was the statistical trend for higher acute GU toxicity in carriers of Thr variant of XRCC3 (ThrThr *plus* ThrMet) compared to MetMet (Fig. 1a) (*p* = 0.087, Fisher exact test) as well as ThrThr vs. MetMet (*p* = 0.058, Fisher exact test). Additionally, PCa patients with ThrThr genotype had higher rate of acute GU toxicity grade ≥ 2. For acute GI toxicity, there was a similar frequency of genotypes of *XRCC3* Thr241Met (Fig. 1b) but contrary to acute GU in GI toxicity MetMet had higher rate of grade ≥ 2. Late GU adverse RT-induced effects were appeared more frequent in patients with ThrThr variant of *XRCC3* while late GI RT-induced effects were appeared more frequent in patients with ThrMet variant. Grade ≥ 2 GU toxicity was the frequent in MetMet while grade ≥ 2 GI toxicity in ThrThr carriers but without statistical significance.

### Radiation-induced CD8 T-lymphocyte apoptosis

RILA levels were measured for 67 out of 88 patients. Median RILA value was 30.24 (mean, 30.65; SD, 13.49; range, 7.96 to 69.86). Kolmogorov–Smirnov test showed that the values of RILA assay have a normal distribution (K–S Dist. = 0.092; *p* = 0.164).

The median value of RILA levels in patients with grade 1 acute GU and GI toxicity was higher than in patients with grade 2 *plus* 3 group but without statistical significance. Also, in patients with late GU toxicity RILA levels were higher in grade 1 compared to grade 2 *plus* 3 group (Table 4). In patients who were analyzed, only four patients experienced late GI toxicity grade 2 and none experienced grade 3. Because of that we did not perform statistical analysis for late GI toxicity and RILA.

**Table 4:** RILA and RT-induced toxicity.

	**RILA number of patient (median; mean; range)**	**Test; *p* value**
**Acute** **GU toxicity**	Grade 1: 41 (31.1; 31.6; 8.0 to 69.9) Grade 2 *plus* 3: 23 (22.8; 29.2; 8.1 to 66.9)	MWU = 524.0; p = 0.467*
**Acute** **GI toxicity**	Grade 1:50 (30.3; 30.9; 8.0 to 69.9) Grade 2: 10 (27.7; 30.7; 15.8 to 47.5)	t (58) = 0.040; *p* = 0.969**
**Late** **GU toxicity**	Grade 1: 29 (33.2; 33.3; 12.2 to 66.9) Grade 2 *plus* 3: 21 (30.1; 31.1; 8.0 to 69.9)	t (48) = 0.527; *p* = 0.600**
	First tertile: Grade 1:9 Grade 2 *plus* 3: 6 Second and third tertile: Grade 1: 20 Grade 2 *plus* 3: 15	χ2_1 =_ 0.016; *p* = 0.900**
**Late** **GI toxicity**	Grade 1: 15 (30.1; 32.3; 13.0 to 69.9) Grade 2: 4 (43.3; 44.2; 40.0–50.3)	t (17) = − 1.485; *p* = 0.156**
	First tertile: Grade 1: 5 Grade 2: 0 Second and third tertile: Grade 1: 10 Grade 2: 4	Fisher exact test *p* = 0.530

*Wilcoxon rank sum test; **t test.

MWU- Mann–Whitney U Statistic.

NA- not applicable.

GU- genitourinary; GI- gastrointestinal.

According to the REQUITE project report, tertile cut-off points could be used to divide the patients into three equal groups of low, mid and high radiation-induced cell death. In our case the first tertile cut-off value was 21.44% and 36.18%. We did not observe a statistically significant difference in the distribution of patients with different radiotoxicity grade among tertiles (Table 4).

### Association of *TGFB1* C-509T, *TGFB1* Leu10Pro, *XRCC1* Arg280His and *XRCC3* Thr241Met with RILA values

In the light of incidence for late GU and GI radiotoxicity, patients with the highest mean value of RILA had TT genotypes for C-509T and ProPro for Leu10Pro *TGFB1* polymorphism. These data reached statistical trend for GU and GI toxicity for C-509T and statistical significance for GI for Leu10Pro polymorphism. For *XRCC3* Thr241Met polymorphism, heterozygotes patients had the highest mean value of RILA but without statistical significance (Table 5).

**Table 5:** Association of polymorphic variants with RILA values.

Polymorphism	Genotype (No of patients): Mean value late GU toxicity RILA	ANOVA	Genotype (No of patients): Mean value late GI toxicity RILA	ANOVA
*TGFB1* C-509T	CC (26): 33.8 CT (17): 27.7 TT (5): 43.0	***p* = 0.080**	CC (12): 32.4 CT (5): 31.4 TT (2): 57.8	***p* = 0.055**
*TGFB1* Leu10Pro	LeuLeu (14): 34.4 LeuPro (24): 29.9 ProPro (8): 35.4	*p* = 0.522	LeuLeu (6): 33.5 LeuPro (10): 30.3 ProPro (2): 57.8	***p* = 0.050**
*XRCC1* Arg280His	NA	NA	NA	NA
*XRCC3* Thr241Met	ThrThr (21): 32.5 ThrMet (18): 35.1 MetMet (7): 27.1	*p* = 0.438	ThrThr (7): 32.7 ThrMet (9): 40.2 MetMet (2): 27.5	*p* = 0.422

NA- not applicable.

Bolded values indicate statistically significant data or statistical trend.

GU- genitourinary; GI- gastrointestinal.

### Logistic regression analysis

According to the concept that minimum ten event for variable is acceptable for logistic regression analysis^19^ and the small number of least frequent outcome in our study (absence of acute GU and GI toxicity- in five and eight patients respectively), we performed logistic regression analysis only for late toxicity.

The logistic regression analysis data were shown in Table 6. Gleason score and risk group were risk factors for late GU while for late GI RT-induced toxicity it was diabetes mellitus type 2. However, in multivariate model Gleason score and risk group (*p* = 0.192 and *p* = 0.367) did not prove to be significant and independent risk factors for late GU toxicity.

**Table 6:** Prediction of late GU and GI radiotoxicity.

Variable	Occurrence of late GU toxicity		Occurrence of late GI toxicity	
	*p* value	OR (95% CI)	*p* value	OR (95% CI)
Age	0.385	1.031 (0.962–1.106)	0.299	1.041 (0.965–1.122)
Initial PSA value	0.610	1.011 (0.970–1.053)	**0.076**	**1.035 (0.996–1.076)**
Diabetes mellitus type 2	0.869	1.104 (0.342–3.567)	**0.001**	**0.145 (0.045–0.466)**
Smoking status	0.871	0.920 (0.340–2.495)	0.634	1.278 (0.466–3.503)
Chronic hypertension	0.983	1.011 (0.394–2.589)	0.721	1.190 (0.457–3.098)
Type of radiotherapy	0.716	0.843 (0.335–2.117)	0.511	0.724 (0.277–1.895)
Gleason score	**0.030**	**2.440 (1.092–5.449)**	0.663	1.158 (0.598–2.244)
Risk group	**0.037**	**2.256 (1.052–4.837)**	0.303	1.497 (0.695–3.227)
RILA value	**0.073**	**1.045 (0.996–1.096)**	0.118	1.033 (0.992–1.075)
*TGFB1* C-509T genotypes	0.670	0.869 (0.455–1.659)	0.867	1.058 (0.544–2.060)
*TGFB1* Leu10Pro genotypes	0.662	1.170 (0.579–2.362)	0.975	0.989 (0.485–2.015)
*XRCC3* Thr241Met genotypes	0.215	0.644 (0.321–1.292)	0.993	0.997 (0.488–2.037)

Bolded values indicate statistically significant data or statistical trend.

GU- genitourinary; GI- gastrointestinal.

## Discussion

A combination of different approaches should be developed to predict patients' individual radiosensitivity, and to reduce side effects of radiation treatment. This will allow clinicians to modify treatment regiments, to improve success and efficacy of the therapy^1^.

Symptoms of GI toxicity can range from a mild increase in bowel movement frequency to more severe complications such as rectal bleeding, pain, or fistula. The acute phase of RT injury is characterized by inflammation in response to therapy, while the late phase is characterized by fibrosis and sclerosis within the GI tract^20^. Urinary toxicity includes the following symptoms: changes in urinary frequency, bladder obstruction, hematuria, urinary incontinence or dysuria^21^.

Irradiation causes direct DNA damage and increases production of free radicals which interact with DNA, thus inducing single-strand or double-strand breaks, and DNA crosslinks ^22^. Cells respond to irradiation by cell cycle arrest thus inducing DNA damage repair. Unrepaired damage causes cell cycle arrest or cell death. Tumors are more susceptible to the DNA damage caused by radiation because of their higher proliferation rates, thus having less time to repair damage than normal tissue^23^. Free radicals induce stress responses, inflammation, and release of cytokines, growth factors, and chemokines. Deficiency in DNA repair genes, cells' ability to undergo apoptosis or impaired cell cycle arrest, as well as changes in genes encoding enzymes involved in free-radical metabolism, and immune response contribute to increased radiosensitivity^23^.

Individual genetic background might be crucial for prediction of normal tissue reaction to RT. In light of that, we tried to find an association between SNPs *TGFB1* C-509T, *TGFB1* Leu10Pro, *XRCC1* Arg280His, and *XRCC3* Thr241Met and RT-induced toxicity of normal tissue.

*XRCC1* gene encodes protein that is involved in single-strand DNA break and base excision repair of radiation-induced damage^11^. XRCC1 fixes base damage and DNA single-strand breaks caused by ionizing radiation interacting with polymerase-beta, DNA ligase III, and poly (ADP-ribose) polymerase^12^. One of candidate biomarker of radiotoxicity could be *XRCC1* Arg280His SNP because of fact that the 280His protein variant is associated with increased repair activity^12^. It has been shown that ArgArg individuals have substantially more chromosome breaks compared to those carrying at least one 280His allele^24^. These findings were supported by the observation that workers exposed to organic solvents carrying the wild type 280 ArgArg had significantly higher levels of chromosomal aberrations than those with one or two variant His alleles^25^.

It has been shown in PCa patients that presence of 280His allele was associated with decreased risk of late toxicity^12^. Other authors have not found any association between late toxicity after RT for treatment of PCa and *XRCC1* Arg280His SNP^26^. Also, in breast cancer, the authors did not observe association of this SNP with fibrosis as late effect of RT or acute skin reaction^27,28^. However, the meta-analysis on 2199 breast cancer patients showed that 280His allele had protective against radiation-induced toxicity^29^. Regarding unexplained role of *XRCC1* Arg280His SNP in normal tissue response to RT, it will require further research. In our study, only 2.4% of patients had ArgHis genotype and everyone else ArgArg. Because of that, we did not perform statistical analysis for *XRCC1* Arg280His polymorphism and RT-induced toxicity. Also, it is important to mention that ArgHis and HisHis were rare variants in some other studies on PCa as well breast cancer patients and were in range 5.1–8.0% and 0–0.9%, respectively^11,26,27^.

*XRCC3* gene encodes protein involved in HR repair of radiation-induced DSBs. During HR repair of DSBs, XRCC3 interacts with RAD51 to promote the initiation of HR and stabilize DNA heteroduplex^30^. One of the most common SNP investigated in *XRCC3* is Thr241Met. The 241Met variant was reported to be associated with elevated levels of DNA adducts^13^, chromosomal deletions^14^, sensitivity to ionizing radiation and cross-linking agents^15^. Thr241Met amino acid substitution may affect the structure of XRCC3 protein and lead to a deficiency in the HR pathway. Consequently, the repair mechanism of DSBs could be moved toward non-homologous end joining, which may result in chromosome instability and the cell’s ability to repair DNA damage lessions^30^. The *XRCC3* Thr241Met was found to be significantly associated with radiation-induced acute skin toxicity and mucositis. Furthermore, *XRCC3* Thr241Met was significantly associated with radiation-induced fibrosis. The presence of *XRCC3* Thr241Met variant was significantly correlated with a higher risk of developing normal tissue reactions to RT after head and neck area and breast irradiation. The absence of significant correlations between this variant and side normal tissue effects was observed after lung or pelvic irradiation^31^.

Contrary to expectations, in our study, there was the statistical trend towards higher acute GU toxicity in carriers of Thr variant. For acute GI toxicity, there was a similar distribution in Thr241Met SNP genotypes. PCa patients with ThrThr genotype had higher rate of acute GU toxicity grade ≥ 2 while in GI toxicity MetMet had higher rate of grade ≥ 2. The late GU toxicity was more common in ThrThr while late GI toxicity in ThrMet patients. The grade ≥ 2 late GU toxicity was more common in MetMet while the grade ≥ 2 late GI toxicity in ThrThr individuals. The possible explanation of these findings is that a combination of unknown genotypes may confer RT-induce toxicity. For example, in PCa patients with the combination ValAla of the antioxidant *SOD2* (superoxide dismutase 2) Val16Ala SNP and ThrMet of *XRCC3* Thr241Met SNP experienced a significant increase in grade 2 late rectal bleeding compared to patients without this particular genotypic arrangement^11^.

It is also interesting to mention that the same polymorphic variant was related to GU but not GI toxicity and vice versa. It indicates that probably exist cell and tissue-specific characteristics that lead to distinct phenotypes upon radiation treatment. Additionally, for different tissue higher grade of toxicity was related to different polymorphic variants. Further study on larger group is necessary to confirm this date and to clarify mechanism underlying this observation.

Fibrosis, as a late toxicity effect of radiation, represents an inflammatory-mediated proliferation response of surviving fibrocytes to growth factors, cytokines, and chemokines released following irradiation^23^. TGFβ1 is a key cytokine associated with inflammation and fibrosis. The presence of the T allele at − 509 bp of the promoter region of *TGFB1* is associated with higher concentrations of TGFβ1 than the C allele^8^. The T>C transition at codon 10 of *TGFB1* results in leucine to proline substitution (Leu10Pro) and in increased levels of TGFβ1 protein^10^.

Initial results from a relatively small Danish patient’s cohort indicated significant associations between the *TGFB1* − 509T and codon 10 Pro allele and increased risk of radiation-induced breast fibrosis^32,33^ but the authors were unable to confirm this association in a subsequent larger study^27^. Data from two British cohorts showed a significant association of the − 509T allele with breast fibrosis following RT^34,35^ as well as later study on early-stage breast cancer patients from MD Anderson Cancer Center (Houston, Texas)^36^. Contrary to previous finding, the studies on nasopharyngeal and breast cancer patients showed an association between 10 Pro allele and lower risk of fibrosis^37,38^. However, a large study on 778 breast cancer patients failed to confirm any association of C-509T and Leu10Pro *TGFB1* and radiation-induced toxicity^39^ as well as further meta-analysis on 2782 participants who received adjuvant breast RT^40^. In PCa, study on 413 patients did not find any association of mentioned *TGFB1* SNPs and late GU and GI toxicity^41^. Damaraju et al. did not find association of C-509T SNP and late rectal and bladder toxicity after RT for prostate^26^ cancer while Peters et al. found that patients with the -509TT genotype had a significantly increased risk of developing late rectal bleeding^7^.

In our study, for late GU toxicity, the CC and CT carriers of C-509T had slightly higher rate than TT. However, heterozygote carriers had nearly double higher rate of grade ≥ 2 toxicity than homozygotes. Contrary to late GU toxicity, individuals with TT genotype had the higher rate of overall late GI toxicity and the heterozygotes had lower rate of grade ≥ 2 than homozygotes. For GI toxicity it is in accordance with some previous data^7^ but for GU toxicity data are missing.

For Leu10Pro SNP in late GU toxicity, LeuLeu and LeuPro carriers had slightly higher rate than ProPro while in late GI toxicity there was a similar frequency of all genotypes of *TGFB1* Leu10Pro. Similar to C-509T for Leu10Pro SNP, heterozygote carriers had higher rate of grade ≥ 2 GU but lower rate of grade ≥ 2 GI toxicity than homozygotes. Our results indicate that different level of TGFβ1 protein is included in late GU in regard to late GI toxicity and the different mechanism that lies behind it. Also, probably different mechanism is at the base of higher grade of toxicity of genitourinary and gastrointestinal tissues.

Although TGFβ1 is a key proinflammatory and profibrotic cytokine, its role in acute toxicity after RT is still unclear. We found that heterozygote carriers of *TGFB1* C-509T had statistically significant lower rate of acute GU and GI toxicity than homozygotes (CC *plus* TT). Additionally, the OR indicated lower risk for acute toxicity development in heterozygote than homozygote PCa patients. The obtained data indicate that CT genotype of *TGFB1* C-509T could be potential biomarkers of lower risk for acute RT-induced toxicity. Additionally, heterozygote PCa patients for *TGFB1* Leu10Pro had lower rate of acute GU and GI toxicity than homozygotes. This is to be expected because of the strong linkage disequilibrium between C-509T and Leu10Pro polymorphisms^39,42^. The obtained results indicated that median dose of TGFβ1 is protective for the development of acute toxicity. The exact mechanism underlining this should be further investigated.

Interestingly, the frequency of acute GU toxicity in grade ≥ 2 arose from CC and CT to TT C-509T genotype while in acute GI toxicity the heterozygote patients had higher frequency of grade ≥ 2 toxicity than CC and TT genotype. For Leu10Pro SNP, the frequency of acute GU and GI toxicity grade ≥ 2 were higher in LeuPro than in homozygote carriers. These data may be explained by different mechanisms of development of RT-induced injury in different tissue or relatively small sample size of PCa patients with grade ≥ 2 toxicity.

PCa clinical variables may contribute to RT-induced toxicity. Because of that, we performed the logistic regression analysis to elucidate these effects. We found that Gleason score and risk group were risk factors for late GU while for late GI RT-induced toxicity it was diabetes mellitus type 2 in univariante model. None of the genotypes of investigated SNPs was shown to be a risk factor for RT-induced toxicity of normal tissue.

Radiosensitivity is typically measured in research studies using patient-derived cells (mainly PBLs or fibroblasts). Cells are irradiated and radiosensitivity can be measured using clonogenic assay, detection of chromosome and DNA damage, cell cycle delay as well as RILA^23,43^. The molecular mechanism linking compromised RILA and late toxicity is still unclear. One could hypothesize that a slow apoptotic response in irradiated lymphocytes promotes cytokine production and attracts inflammatory immune cells that could be at the origin of late toxicities^43^.

According to the REQUITE project report, tertile cut-off points could be used to divide the patients into three equal groups of low, mid and high RILA. Although, the mean value of RILA in grade 1 was slightly higher than in grade 2 *plus* 3 group for acute GU and GI toxicity as well as late GU toxicity, we did not observe statistically significant correlation. For late GI toxicity the mean value of RILA in patients with grade 1 was lower than in patients with grade 2 *plus* 3. This could be explained by fact that we only had four patients in group ≥ 2 toxicity and according to the extremely small sample size we did not perform statistical analysis. Additionally, our tertile cut-off was slightly higher than those in REQUITE project study.

Previous multicenter studies showed a good negative predictive value in patients with high RILA values and low-grade late toxicity following RT^16,17^. However, the first study of Ozsahin et al. (2005) included 399 patients from different cancer cite and there were only 36 of PCa patients among them. We hypothesize that CD4 and CD8 lymphocytes from blood of patients with different cancer should react differently to radiation and the chemotherapy and hormone therapy maybe have some impact on radiation response. The other study of Azria et al. related RILA to RT-induced fibrosis^17^. One of recent study that included 245 patients with PCa showed that RILA does not correlate with the inter-individual variation in maximal late GU or GI toxicity after radiation^44^. In the same manner, the REQUITE study showed that the RILA assay is a very robust marker for fibrosis (breast or head and neck fibrosis) after RT. The assay seemed less robust with other types of toxicity like vascular damage lesions, which could explain why the correlation is not as good as in our study. In the REQUITE project, the toxicity endpoints were changes in breast appearance at 2 years measured using photographs, pneumonitis at 1 year and rectal bleeding at 2 years. These primary endpoints were selected because they show radiation dose–response relationships.

We found correlation of *TGFB1* variants (TT for C-509T and ProPro for Leu10Pro) and highest mean value RILA for late GU and GI radiotoxicity as statistical trend in case of C-509T and significance in Leu10Pro for GI radiotoxicity. For *XRCC3* Thr241Met, heterozygotes patients had the highest but not statistically significant mean value of RILA. But these data should be taken with caution for GI toxicity given the small number of patients with GI toxicity, particularly for TT and ProPro *TGFB1* and MetMet *XRCC3* with only two patients each. Furthermore, the study by Azria et al. showed that high risk of late radiotoxicity was associated with a low RILA values and the presence of four or more SNPs in selected genes (*ATM, SOD2, TGFB1, XRCC1, XRCC3*, and *RAD2*)^45,46^. Combining determination of RILA and profiling of a specific set of SNPs in genes implicated in radiosensitivity might represent a promising strategy for identification of cancer patients at high risk of late normal tissue reactions after RT, individual RT optimization, and improvement of patients’ quality of life. Complex genetic and biological factors underlying associations between RILA and late radiotoxicity in patients with cancer should be further explored^18^. The large multicentric studies for better defining cut-off RILA values for each cancer type are also needed.

## Conclusions

We found that CT genotype of *TGFB1* C-509T SNP could be potential protective biomarker for acute GU as well as GI RT-induced toxicity while Thr variant of *XRCC3* Thr241Met SNP could identify patients at risk for acute GU toxicity. For other investigated polymorphisms we did not observe any association with acute or late adverse effects of RT. Also, we did not show correlation of RILA value with RT-induced normal tissues injury. Further studies on larger study groups are needed to confirm our results.

The finding combination of assays that are characterized by a high level of sensitivity and specificity to predict susceptibility to the development of adverse effect of RT will serve as a powerful tool in precision medicine approach. This approach might contribute to the development of tests for the prediction of individual radiosensitivity, and even RT-induced secondary malignancies, not only for RT for prostate cancer but also for the prediction of other radiation-induced adverse effects in other malignancies treated with RT, such as breast cancer, brain malignancies, lung cancer, etc.

## Methods

### Study population

Eighty-eight patients who had a histologically confirmed localized or locally advanced PC were included in the study. Patients were treated with 3D conformal RT (3DCRT) (n = 76) or Volumetric Modulated Arc Therapy (VMAT) (n = 12) at the Institute for Oncology and Radiology of Serbia from January 2016 to August 2019 with radical (72 Gy in 36 fractions) (in 50 patients) or postoperative/salvage (66 Gy in 33 fractions) (38 patients) RT without previous hormonal therapy. The details of the study were explained to the patients and informed consent forms were signed by the participants. The study protocol was approved by the Ethics Committee of the Institute of Oncology and Radiology of Serbia (approval No 3348/1–01). The study was carried out according to the principles of the Declaration of Helsinki.

Acute and late radiotoxicity were evaluated according to RTOG/EORTC Radiation Morbidity Scoring Criteria modified by Peeters^47^. Side-effects occurring within 120 days from the start of RT were considered as acute radiation morbidity. The radiation oncologist recorded baseline symptoms and acute symptoms during RT and at first control (30 days after the end of RT). Late toxicity was scored from 120 days after the start of treatment. All clinical data and blood samples were prospectively collected. Exclusion criteria were as follows: chronic infective diseases, neoadjuvant or concomitant hormonal therapy, the presence of enlarged lymph nodes (N1 stage) at the time of diagnosis, the presence of distant metastasis (M1 stage), Karnofsky index < 80, and previous pelvic irradiation.

### Peripheral blood mononuclear cell and DNA extraction

Peripheral blood mononuclear cells (PBMC) were extracted with Histopaque-1077 (Sigma-Aldrich, USA) solution according to the manufacturer’s instructions. DNA from PBMC was extracted by salting out method^48^.

### Genotyping of *TGFB1*, *XRCC1* and *XRCC3* polymorphisms

**Figure 2:**
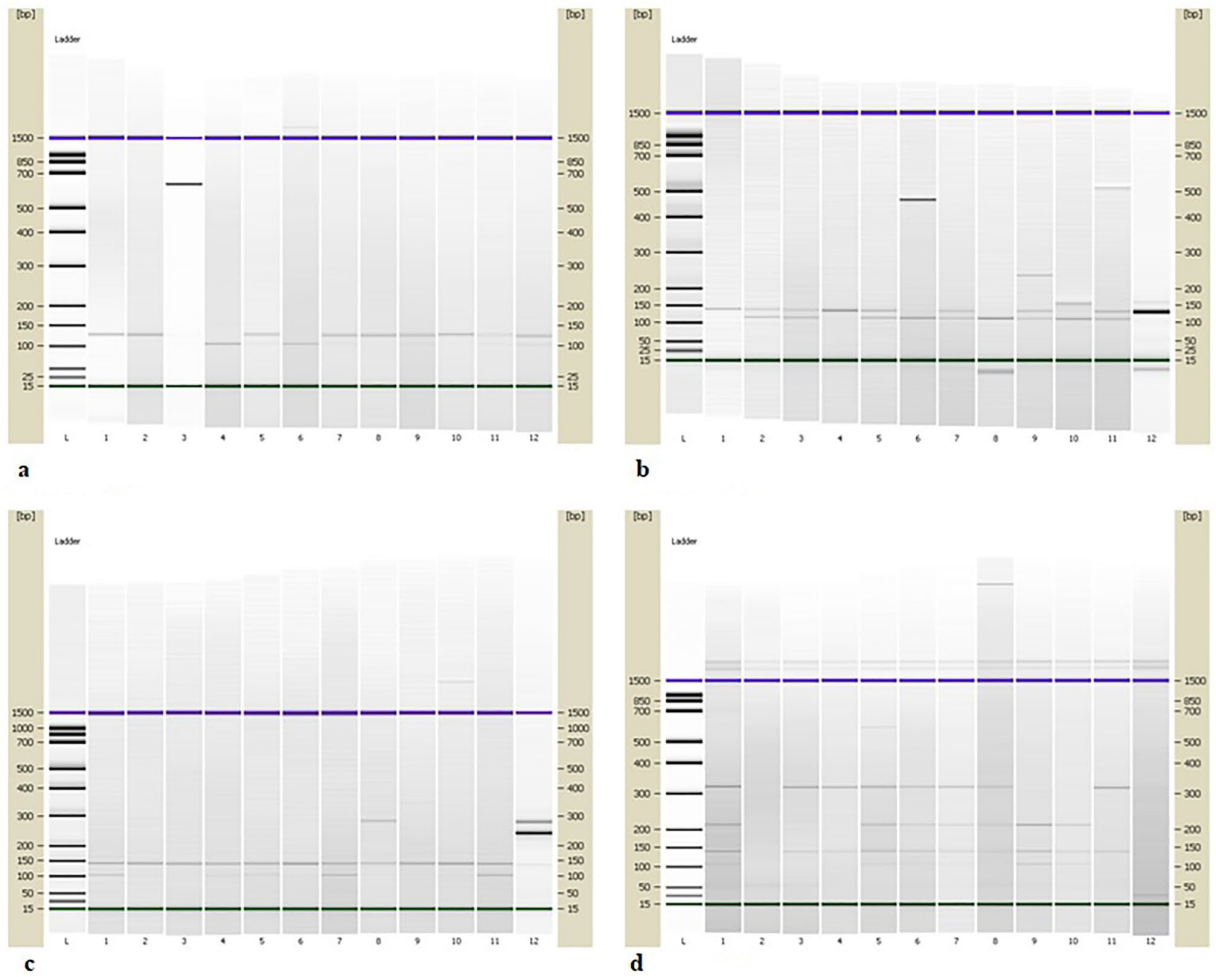
Detection of polymorphisms by PCR–RFLP method. (**a**) *TGFB1* C-509TC: column L- *DNA Ladder*; column 1, 2, 8, 10- CC genotype; column 5, 7, 9, 11, 12- CT genotype; columns 4, 6- TT genotype. (**b)** *TGFB1* Leu10Pro: column L- *DNA Ladder*; columns 1, 4- ProPro genotype; column 2, 3, 5, 7, 9, 11- LeuPro genotype; columns 6, 8, 10- LeuLeu genotype, column 12- PCR product. (**c)** *XRCC1* Arg280His: column L- *DNA Ladder*; columns 1, 2, 3, 4, 5, 6, 7, 9, 10, 11- ArgArg genotype; column 8- ArgHis genotype; column 12- PCR product. (**d)** *XRCC3* Thr241Met: column L- *DNA Ladder*; columns 1, 5, 6, 7, 8- ThrMet genotype; columns 3, 4, 11- ThrThr genotype; columns 9, 10- MetMet genotype.

The polymorphic variants of promoter C-509T (rs1800469) and Leu10Pro (rs1800470) of *TGFB1*, Arg280His (rs25489) of *XRCC1*, and Thr241Met (rs861539) of *XRCC3* were determined by polymerase chain reaction-restriction fragment length polymorphism (PCR–RFLP) analysis. The DNA regions around mentioned SNPs in *TGFB1*, *XRCC1*, and *XRCC3* genes were amplified, separately, in 25 μL PCR reaction volume contained 12.5 μL DreamTaq PCR Master Mix 2x (Thermo Fisher Scientific, USA), 200 ng of genomic DNA, and 0.4 µM of each sense and antisense primers (Invitrogene, USA). Primers sequences and PCR conditions were given in Table 7^49–51^. PCR products of *TGFB1*, *XRCC1*, and *XRCC3* were visualized by electrophoresis on 2% agarose gel and concentrations were measured on NanoDrop (Shimadzu, Japan). PCR products were digested by BspTI, PvuII, RsaI and FatI fast digest restriction enzyme (Thermo Fisher Scientific, USA), respectively. After digestion, the fragments were separated on 2100 Bioanalyzer (Agilent, USA) using DNA 1000 kit according to the manufacturer’s instructions. The length of PCR products and fragments were given in Table 8. Figure 2a–d show electropherograms from 2100 Bioanalyzer which contain DNA fragments obtained by PCR–RFLP method.

**Table 7:** Primers’ sequences and PCR conditions.

Gene	SNP	Primers (5′–3′)	PCR conditions
*TGFB1*	C-509T	F:GTCGCAGGGTGTTGAGTGACAG R:AGGGGGCAACAGGACACCTTA	95 °C, 5 min- initial denaturation, 95 °C, 30 s; 62 °C, 45 s; 72 °C, 1 min- 35 cycles, 72 °C, 10 min- final elongation
*TGFB1*	Leu10Pro	F:CTCCGGGCTGCGGCTGCAGC R:GGCCTCGATGCGCTTCCGCTTCA	95 °C, 5 min- initial denaturation, 95 °C, 30 s; 62 °C, 45 s; 72 °C, 1 min- 35 cycles, 72 °C, 10 min- final elongation
*XRCC1*	Arg280His	F:TGGGGCCTGGATTGCTGGGTCTG R:CAGCACCACTACCACACCCTGAAGG	94 °C, 5 min- initial denaturation, 94 °C, 40 s; 55 °C to 69 °C, 40 s; 72 °C, 30 s- 35 cycles, 72 °C, 10 min- final elongation
*XRCC3*	Thr241Met	F: GGTCGAGTGACAGTCCAAAC R: TGCAACGGCTGAGGGTCTT	95 °C, 5 min- initial denaturation, 95 °C, 1 min; 60 °C, 1 min; 72 °C, 1 min- 40 cycles, 72 °C, 5 min- final elongation

**Table 8:** The length of PCR products, restriction enzymes and fragments.

Gene	SNP	Restriction enzymes	PCR products (bp)	Fragments size (bp)
*TGFB1*	C-509T	BspTI	123	CC: 123 CT: 123, 101,22 TT: 101, 22
*TGFB1*	Leu10Pro	PvuII	136	CC (ProPro): 136 CT (LeuPro):136, 117, 19 TT (LeuLeu): 117, 19
*XRCC1*	Arg280His	RsaI	280	GG (ArgArg): 140 GA (ArgHis): 280, 140 AA (HisHis): 280
*XRCC3*	Thr241Met	FatI	456	CC (ThrThr): 315, 141 CT (ThrMet): 315, 210, 141, 105 TT (MetMet):210, 141, 105

### RILA assay

Before RT a single blood sample was collected from each patient in a 4-ml BD Vacutainer lithium heparin tubes. The whole blood samples were diluted 1:10 in RPMI 1640 containing 20% fetal bovine serum, irradiated at 0 and 8 Gy, and incubated for 48 h. The cells were then labeled with FITC-conjugated anti-CD8 monoclonal antibodies, red blood cells were lysed, and the DNA of the remaining cells stained with propidium iodide. Samples were measured using a FACScan flow cytometer, and data analysis was done using Kazula software (Beckman Coulter, USA). Apoptotic CD8 T-lymphocytes were defined as those cells staining positively for their cell type-specific antibodies and displaying reduced propidium iodide fluorescence and cell size. Data from at least 10,000 cells per sample were acquired^45^. The addition of CD4 into the model did not contribute significantly in separating the two groups^16^. Therefore, we took into account only CD8 results for the present study.

### Statistical analysis

Deviations of the genotype frequencies of *TGFB1* C-509T and Leu10Pro, *XRCC1* Arg280His, and *XRCC3* Thr241Met polymorphisms from those expected under Hardy–Weinberg equilibrium in the studied groups were assessed using the χ2 test.

The differences in the distribution of genotypes of *TGFB1* C-509T and Leu10Pro, *XRCC1* Arg280His, and *XRCC3* Thr241Met between patients with or without acute or late RT-induced GU or GI toxicity, as well as different grade of toxicity and RILA were tested by χ2 and Fisher’s exact test. Kolmogorov–Smirnov test was used to test normality of RILA assay date. To test the differences in RILA value between the groups with or without toxicity as well as different grade of toxicity t-test or Wilcoxon rank sum test were used depending on the distribution of data in groups. The RILA data in different polymorphic variants of *TGFB1*, *XRCC1,* and *XRCC3* were compared by one way ANOVA test. *p* Values ≤ 0.05 were considered statistically significant, while *p* values between 0.1 and 0.05 were pointed out as a statistical trend. The genotype-specific risks were estimated as odds ratios (OR) with associated 95% confidence intervals (CIs) for dominant, recessive, codominant, over-dominant genetic model in case of statistically significant distribution between groups were observed^52^. Positive OR (OR > 1) with 95% CI that does not overlap the null value of OR = 1 was considered statistically significant.

For evaluation of the relationship of the outcome dependent variable (RT-induced toxicity) with potential predictors (clinical-pathological characteristics, polymorphisms and RILA) logistic regression analysis was used. The models were computed both as univariate for all predictors and multivariate for selected predictors that reached *p* < 0.05 in univariate analysis. Results of statistical evaluations were accompanied by OR with 95% CIs. Data were analyzed by SigmaStat 3.5 and MedCalc.

## Data Availability

The data that support the findings of this study are not openly available due to [reasons of sensitivity e.g. human data] and are available from the corresponding author upon reasonable request.
